# Fortuitously detected primary ovarian carcinoid tumor: A case report

**DOI:** 10.1097/MD.0000000000034391

**Published:** 2023-08-04

**Authors:** Hwa Yeon Choi, Min Gyoung Pak, Jung-Woo Park

**Affiliations:** a Department of Obstetrics and Gynecology, Dong-A University College of Medicine, Busan, Republic of Korea; b Department of Pathology, Dong-A University College of Medicine, Busan, Republic of Korea.

**Keywords:** carcinoid tumor, ovary, case reports

## Abstract

Carcinoid tumors, derived from the cells of the disseminated neuroendocrine system, are rare, slow-growing neuroendocrine neoplasms that display a relatively indolent disease course. The majority of carcinoids are found within the gastrointestinal tract and bronchopulmonary system. Primary ovarian carcinoids are rare and account for merely 1% of all carcinoid tumors. We describe our experience of a rare case of primary ovarian carcinoid, presenting as chronic constipation, with no other carcinoid symptoms such as flushing, diarrhea, and wheezing.

**Patient concerns:**

A 51-year-old postmenopausal woman with chronic constipation visited the clinic for routine check-up of her preexisting uterine fibroids. She had undergone hemorrhoidectomy 3 years ago. Physical examination revealed a soft abdomen without direct or rebound tenderness. Transvaginal ultrasonography revealed two subserosal fibroids, which had increased in size compared with previous ultrasonographic findings. A 3 cm hyperechoic mass was also detected in the right ovary. Her blood and urine tests were unremarkable, with no ascites in the pelvic cavity. She had a normal CA-125 level of 5.5 units/mL.

**Diagnosis, interventions, and outcomes:**

The patient underwent a robot-assisted hysterectomy and right salpingo-oophorectomy because of enlarging fibroids and the right ovarian mass. Subsequently, based on the pathological and immunohistochemical findings, she was diagnosed with a primary ovarian carcinoid. The mass consisted of the insular and trabecular types of tumor cells. It was positive for pan-cytokeratin and synaptophysin, and the Ki-67 proliferation index was less than 1%. A follow-up positron emission tomography-computed tomography revealed no distant metastasis. Six months postoperatively, the patient was doing well without any signs of recurrence.

**Lessons:**

Primary ovarian carcinoids without teratoma components are rare. It is crucial to make an accurate diagnosis based on the immunohistochemical staining results. Diagnosis in the early stages of the disease are associated with a favorable prognosis, but regular follow-up is mandatory.

## 1. Introduction

Primary ovarian carcinoids are uncommon, and they account for 1% of all carcinoid tumors.^[1]^ Carcinoid tumors, derived from the cells of the disseminated neuroendocrine system, are rare, slow-growing neuroendocrine neoplasms that display a relatively indolent disease course. They most often occur in the lungs, small intestine, and rectum.^[2]^ The incidence and prevalence of carcinoid tumors have increased largely due to an increase in the diagnostic rate with the development of more sensitive diagnostic techniques.^[2]^ However tumors originating from the genital tract are still rare.^[2,3]^ This study presents a case of an incidentally detected primary ovarian carcinoid tumor.

## 2. Ethics approval and consent for publication

Ethics approval was not applicable as this was a retrospective case study.

Written informed consent was obtained from the patient for publication of this case report and any accompanying images. A copy of the written consent is available for review by the Editor-in-Chief of this journal.

## 3. Case presentation

A 51-year-old postmenopausal woman visited the clinic for routine checkup of her preexisting uterine fibroids. She had chronic constipation since the last 20 years, occasionally requiring enema or laxative. She had undergone hemorrhoidectomy 3 years ago. Her family history was unremarkable. Physical examination revealed a soft abdomen without direct or rebound tenderness. Transvaginal ultrasonography revealed 2 subserosal fibroids, measuring 7.7 and 5.5 cm, respectively, which had increased in size when compared with previous ultrasonographic findings. A 3 cm hyperechoic mass was also detected in the right ovary. No ascites was noted in the pelvic cavity, and the blood and urine tests were unremarkable. She had a normal CA-125 level of 5.5 U/mL.

Intraoperatively, multiple fibroids and a right ovarian mass were identified. The surface of the ovarian tumor was white to yellow without gross abnormalities. Robot-assisted hysterectomy and right salpingo-oophorectomy were performed. The patient recovered and was discharged on postoperative day 3.

**Figure 1.**
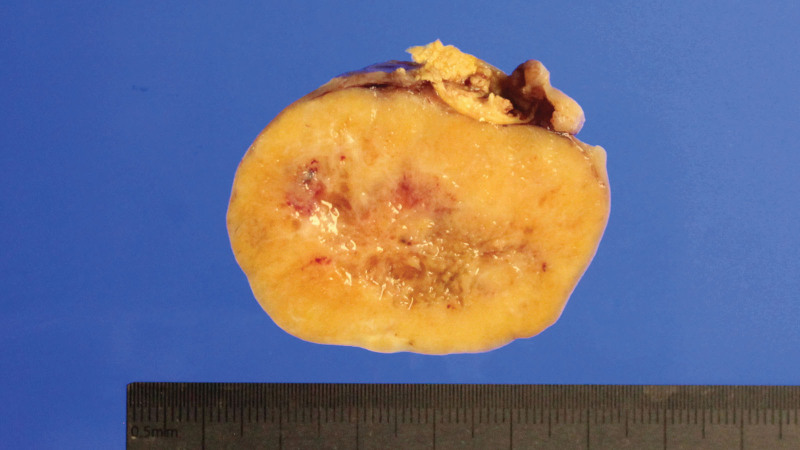
Cross-section of the right ovarian mass.

**Figure 2.**
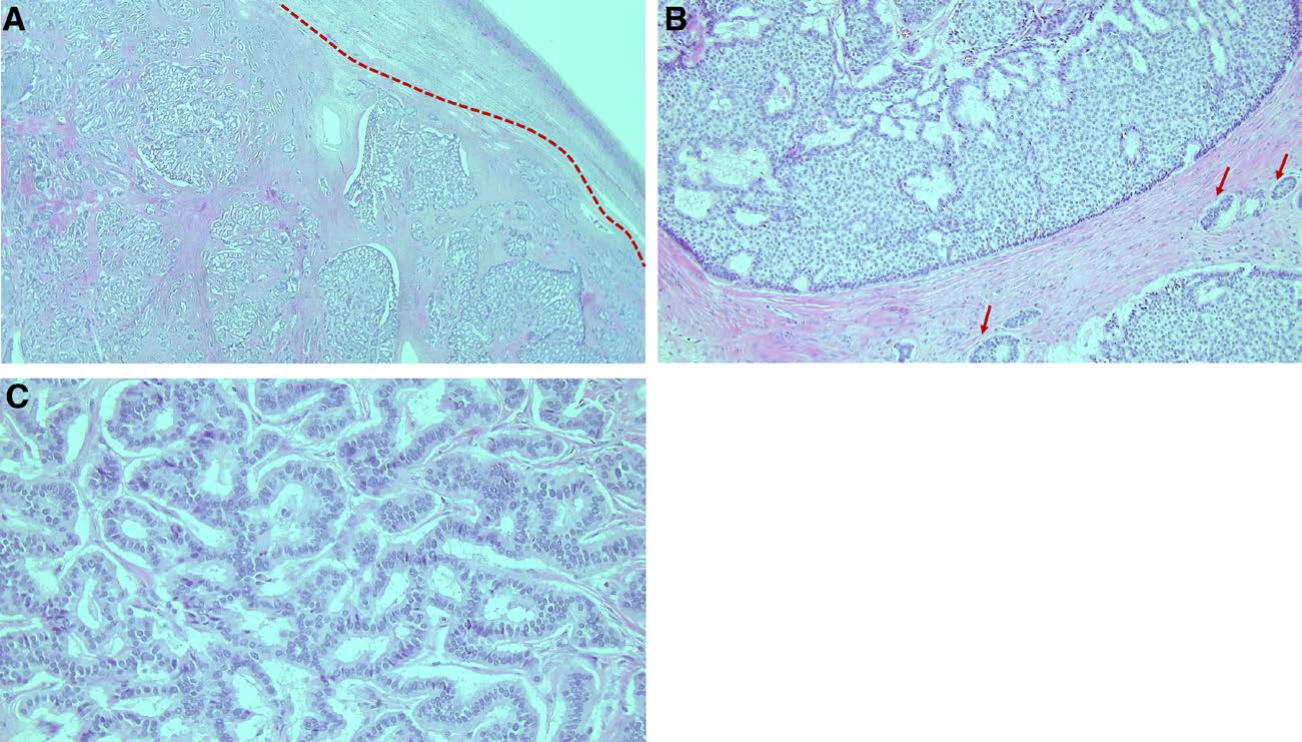
Microscopic (histologic) findings of the tumor cells with hematoxylin-eosin staining. (A) The tumor cell nests were surrounded by thick fibrotic stroma (below the dot line). There were no hemorrhagic or necrotic foci (×20 magnification). (B) The tumor exhibited mixed growth patterns, predominantly the trabecular but also focally insular types (arrows) (×100 magnification). (C) The trabecular-type tumor cells were homogeneously round to oval with pink cytoplasm. No mitotic figures were noted (×200 magnification).

**Figure 3.**
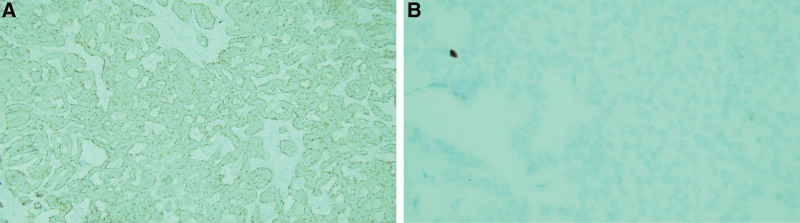
Immunohistochemical staining of tumor cells. (A) The tumor cells exhibited diffuse membranous positivity for synaptophysin (×100 magnification). (B) The Ki-67 proliferation index of <1% was consistent with that of a carcinoid tumor (×400 magnification).

On gross examination, it was observed that a well-circumscribed oval mass, measuring 5 × 4 cm had completely obliterated and taken over the right ovary. The ovarian capsule was intact and tumor-free (Fig. 1). Microscopic examination of the oval mass revealed tumor cell nests, surrounded by thick fibrous stroma, exhibiting mixed growth patterns, particularly of the trabecular and focal insular types. There were no hemorrhagic or necrotic foci. The tumor cells were homogeneously round to oval with pink cytoplasm. No mitotic figures were noted on the hematoxylin-eosin stain (Fig. 2). Immunohistochemistry results revealed dot-like pan-cytokeratin positivity and diffuse strong membranous synaptophysin positivity. The Ki-67 proliferation index was <1%, which was consistent with that of a carcinoid tumor (Fig. 3).

After establishing the pathological diagnosis, the patient underwent a positron emission tomography-computed tomography to evaluate for distant metastasis. There was no evidence of distant metastasis. Six months postoperatively, the patient was doing well without any signs of recurrence.

## 4. Discussion

Primary ovarian carcinoids account for only 0.1% of all ovarian neoplasms.^[1,4]^ They are the most common primary neuroendocrine tumors in the genital tract.^[5]^ The cause of primary ovarian carcinoids is unclear, but it originates from germ cells.^[3]^ They are generally associated with mature cystic teratoma, but a pure form can also occur independently.^[5,6]^ The age at diagnosis varies from 14 to 83 years, and peri- and postmenopausal women are the most affected.^[1]^ Patients with primary ovarian carcinoids may present with no or nonspecific symptoms. Georgescu et al^[7]^ analyzed 99 cases of primary ovarian carcinoids, described in 68 published articles and found that approximately 70% of the patients were symptomatic. In particular, the presenting symptoms were carcinoid heart disease in 29%, an abdominal mass in 17%, constipation in 12%, and abnormal uterine bleeding in 7%.^[7]^ Matsuda et al^[8]^ reported a case of ovarian strumal carcinoids, presenting as constipation, induced by the peptide YY hormone, which was secreted by the tumor, resulting in impaired intestinal motility.

Primary ovarian carcinoids are typically detected as adnexal masses during physical examination or ultrasonography. They are also incidentally detected intraoperatively. Pathologically, the masses are categorized into the insular, trabecular, mucinous, stromal, and mixed types.^[6]^ The insular type is the most common type of primary ovarian carcinoids. It is microscopically composed of a nest of polygonal cells with round or oval hyperchromatic nuclei.^[3]^ The trabecular-type is composed of parallel cells, arranged in a ribbon-like pattern, within a fibrous stroma.^[5]^ Immunohistochemistry is essential in the diagnosis of primary ovarian carcinoids. The classic neuroendocrine markers are synaptophysin and chromogranin. CD56 is also useful but not specific because it is also expressed by sex cord-stromal tumors or adenocarcinomas of the ovary.^[3]^ The Ki-67 proliferation index is typically <1% in insular, trabecular, and stromal types of primary ovarian carcinoids.^[5]^

For treatment of primary ovarian carcinoids, the patient’s age, histological type, and stage should be considered.^[1]^ Most primary ovarian carcinoids are often unilateral and confined within the ovary. Early-stage primary ovarian carcinoids are curable with surgery alone, but it is necessary to rule out gastrointestinal metastasis.^[3,9]^ Positron emission tomography-computed tomography is also useful for assessing the extent of the tumor growth and for distinguishing between primary and metastatic tumors.^[3]^ Chemotherapy is considered for the treatment of advanced-stage tumors.^[1]^ Stage I disease is associated with a favorable prognosis with a 10-year survival rate of almost 100%. By contrast, the advanced-stage disease is associated with a poor prognosis and a 5-year survival rate of only 33%.^[4]^

In the present case, the patient had a mixed tumor, consisting of the insular and trabecular types, without a teratoma component. Hence, this case can be considered as rare since most ovarian trabecular-type carcinoids were accompanied by teratoma. The histologic diagnosis of the tumor was confirmed by the positive immunohistochemical staining for pan-cytokeratin, synaptophysin, and Ki-67. The patient had chronic constipation, but there were no other carcinoid symptoms, such as flushing, diarrhea, and wheezing.

## 5. Conclusions

Most patients with primary ovarian carcinoids are asymptomatic. Despite the development of more sensitive diagnostic techniques such as computed tomography, preoperative detection can be difficult, and the final diagnosis of primary ovarian carcinoids is based on pathological examinations. Thus, an accurate diagnosis should be established based on the immunohistochemical findings. Early-stage primary ovarian carcinoids have a low malignant potential and favorable prognosis. However, regular follow-up examinations are mandatory.

## Author contributions

**Conceptualization:** Hwa Yeon Choi, Min Gyoung Pak, Jung-Woo Park.

**Funding acquisition:** Hwa Yeon Choi.

**Investigation:** Hwa Yeon Choi.

**Methodology:** Hwa Yeon Choi.

**Project administration:** Jung-Woo Park.

**Supervision:** Jung-Woo Park.

**Visualization:** Hwa Yeon Choi, Min Gyoung Pak.

**Writing – original draft:** Hwa Yeon Choi, Min Gyoung Pak.

**Writing – review & editing:** Hwa Yeon Choi, Min Gyoung Pak, Jung-Woo Park.

## References

[1] Zhai LR Zhang XW Yu T. Primary ovarian carcinoid: two cases report and review of literature. Medicine (Baltimore). 2020;99:e21109. 33019380 10.1097/MD.0000000000021109 PMC7535635

[2] Dasari A Shen C Halperin D. Trends in the incidence, prevalence, and survival outcomes in patients with neuroendocrine tumors in the United States. JAMA Oncol. 2017;3:1335–42. 28448665 10.1001/jamaoncol.2017.0589 PMC5824320

[3] Reed NS Gomez-Garcia E Gallardo-Rincon D. Gynecologic Cancer InterGroup (GCIG) consensus review for carcinoid tumors of the ovary. Int J Gynecol Cancer. 2014;24(suppl 3):S35–41. 25341578 10.1097/IGC.0000000000000265

[4] Yan F Zhou Q Lin Y. Clinicopathological analysis of primary carcinoid tumour of the ovary arising in mature cystic teratomas. J Int Med Res. 2021;49:030006052110346. 10.1177/03000605211034666 PMC8408898 34459278

[5] Howitt BE Kelly P McCluggage WG. Pathology of neuroendocrine tumours of the female genital tract. Curr Oncol Rep. 2017;19:59. 28735441 10.1007/s11912-017-0617-2

[6] Bai X Li N Wang F. Primary ovarian trabecular carcinoid tumor: a case report and literature review. Arch Gynecol Obstet. 2010;282:407–11. 20652280 10.1007/s00404-010-1600-4

[7] Georgescu T-A Bohiltea RE Varlas V. A 15-year comprehensive literature review of 99 primary ovarian carcinoid tumors. Clin Exp Obstet Gynecol. 2022;49:16.

[8] Matsuda K Maehama T Kanazawa K. Strumal carcinoid tumor of the ovary: a case exhibiting severe constipation associated with PYY. Gynecol Oncol. 2002;87:143–5. 12468356 10.1006/gyno.2002.6785

[9] Pang L Guo Z. Primary neuroendocrine tumors of the ovary: management and outcomes. Cancer Med. 2021;10:8558–69. 34773393 10.1002/cam4.4368 PMC8633223

